# The nexus between urban green space, housing type, and mental health

**DOI:** 10.1007/s00127-022-02266-2

**Published:** 2022-04-11

**Authors:** Xiaoqi Feng, Renin Toms, Thomas Astell-Burt

**Affiliations:** 1 School of Population Health, Faculty of Medicine, University of New South Wales, Sydney, Australia; 2 School of Health and Society, Faculty of Arts, Social Sciences, and Humanities, University of Wollongong, Wollongong, Australia; 3 Population Wellbeing and Environment Research Lab, PowerLab, Sydney, Australia

**Keywords:** Mental health, Housing type, Green space, Tree canopy, Open grass

## Abstract

**Introduction:**

Momentum for urban densification is increasing opportunities for apartment-living, but can result in reduced green space availability that negatively influences mental health. However, in contexts where apartment-living is atypical and commonly viewed as secondary to house-ownership, it may be a stressful antecedent condition (or marker of selective processes aligned with psychological distress) wherein occupants could benefit disproportionately from green space.

**Method:**

Data were extracted from the Sax Institute’s 45 and Up Study baseline (2006–2009, *n = *267,153). The focus was on subsets of 13,196 people living in apartments and 66,453 people living in households within the cities of Sydney, Newcastle and Wollongong. Multilevel models adjusted for confounders tested associations between psychological distress (Kessler 10 scale) with percentage total green space, tree canopy and open grass within 1.6 km road network buffers.

**Results:**

Psychological distress was higher in occupants of apartments (11.3%) compared with houses (7.9%). More green space was associated with less psychological distress for house-dwellers (OR = 0.94, 95% CI = 0.91–0.98), but there was no association for apartment-dwellers. More tree canopy was associated with lower psychological distress for house-dwellers (OR = 0.88, 95% CI = 0.85–0.92) and apartment-dwellers (OR = 0.87, 95% CI = 0.79–0.96). Open grass was associated with more psychological distress among house-dwellers (OR = 1.06, 95% CI = 1.00–1.13) and also for apartment-dwellers (OR = 1.20, 95% CI = 1.07–1.35).

**Conclusions:**

Overall, investments in tree canopy may benefit the mental health of house and apartment residents relatively equally. Urban tree canopy in densely populated areas where apartments are common needs to be protected. Further work is needed to understand factors constraining the prevention potential of open grass, to unlock its benefits for mental health.

## Introduction

Increasing urban green space is currently a widely promoted policy option supported by evidence of health benefits [1]. Restoration of mental health is widely understood to be one of three inter-related domains of pathways [2] for explaining inverse association between green space and morbidity or mortality [3]. Mental health benefits may be induced through physical, visual and/or olfactory means of contact with green space, which often interact with social connection, physical activity and mitigation of ambient hazards (e.g., heatwaves) for stress relief and renewal of depleted cognitive capacities [4].

However, consistent magnitude and direction of association between green space and health is not always observed [5]. Mixed results may be partly due to contextual contingencies operating at the scale of the individual, community and city, [6–9] fuelling variation in the potency of underlying mechanisms. Theories of stress reduction [10] and attention restoration [11] both hypothesise benefits from green space emerge within the context of a stressful antecedent condition, such as a stressful life experience. Emerging research on health inequalities indicate persons in disadvantaged circumstances, who are known to be at a higher risk of stress, [12] may benefit more from contact with green space [13]. But what if aspects of urban fabric, or the societal connotations of it, not only impose a psychosocially taxing condition, but also further constrain salutogenic effects of green space among its residents?

This study addresses this contingency of association between green space and mental health within the context of housing type. In some societies such as Australia where detached houses have been the norm for decades, apartment-living has been described as a stressful experience due to a range of stigmatising attitudes ranging from perceived negligent parenting, to social deviancy [14]. Apartment-dwellers are often subject to conditions that deprive them of companionship with dogs and, in many cases, the built environment may be antisocial too due to a lack of private garden. Further stress may be added due to financial insecurity, with many persons selecting into apartments at high financial cost to access desirable locations (e.g., prestigious schools), where houses can be unaffordable [15]. This may mean residents spend more time working to afford high rents than enjoying local green spaces.

We propose to test duelling hypotheses using data in Australia. On one hand, apartment residents may benefit disproportionately in comparison to their peers in houses due to the abovementioned stressful antecedent conditions. On the other hand, the same antecedent conditions may constrain apartment residents’ interactions with green space, limiting the net impact on their mental health. We examined these hypotheses with respect to the availability of green space in general, as well as tree canopy and open grass separately, given previous work reporting contrasting results for mental health and green space type [16–18].

## Method

This study utilised data from the Sax Institute’s 45 and Up Study [19], which is Australia’s largest ongoing study of health and ageing. Participants were recruited into the baseline survey of the 45 and Up Study from the Services Australia (formerly the Department of Human Services and Medicare Australia) enrolment database between 2006 and 2009 with a response of about 18% (*n = *267,153). The 45 and Up Study’s demographic profile at baseline are generally representative of the Australian population of the same age [20]. Written informed consent for data linkage was provided by all participants. Data collection was approved by the University of New South Wales Human Research Ethics Committee (HREC). This study was approved by the University of Wollongong HREC and the NSW Population and Health Services Research Ethics Committee.

We restricted the sample to persons living in houses (*n = *66,453) or apartments (*n = *13,196) located in the coastal cities of Sydney, Wollongong or Newcastle (subset using the Australia Bureau of Statistics ‘Urban Centres and Localities’ boundaries). Sydney, Wollongong and Newcastle are the major urban centres in the state of New South Wales (NSW), at 4,321,535, 261,896 and 322,278 residents, respectfully, according to the 2016 Census. These housing types were by far the most populous in the sample, with aged care as the only other housing type to reach over 500 participants (approximately 2400). However, given the strong health-related selection [21–23] of people into aged care in Australia, these participants were omitted along with those in other much smaller categories (e.g., hostels, mobile homes).

The sample was also restricted to those with complete data for the Kessler 10 (‘K10’) psychological distress scale [24]. The K10 is a widely used self-reported 10-item measure covering topics, such as anxiety, depressive symptoms, hopelessness and restlessness (see Appendix 1). Summed scores ≥ 22 are indicative of a high risk of psychological distress experienced over the previous 4 weeks [25].

Three green space measures (total green space, tree canopy, open grass) were derived from high resolution (2 m) land-use data from Pitney Bowes Ltd for 2016. Tree canopy included street trees, trees on public and private property, deciduous and evergreen. Open grass includes all grass not obscured by tree canopy and as such, underestimates the total grass coverage. Total green space included tree canopy, open grass, and also other low-lying vegetation (shrub) which constituted a minority vegetation land-use [26–28]. Percentage green space variables were constructed using a road-network buffer around centroids of Mesh Blocks of residence of 1.6 km, representing a reasonable walking distance [29]. This aligned with a ‘cumulative opportunities’ approach to measuring the quantity of green space available within designated catchments, [30] as opposed to the more restrictive travel distance to the nearest green space that would ignore alternative settings that may also be beneficial. Each green space variable was measured in intervals relevant to urban planning policies in various cities, [31–34] for example, urban tree canopy cover at < 10%, 11–20%, 21–30%, and ≥ 30%. Each green space variable was tested separately.

Multilevel logistic regressions stratified by housing type in R were used to test association between green space and psychological distress for people in houses or in apartments separately. Random intercepts were fitted on statistical area 3 geographical boundaries, constructed by the Australian Bureau of Statistics, which helped to account for variations in mental health within and between the three cities. All models were adjusted for competing explanations for association between green space and mental health, including sex (female, male), age (45–54 years, 55–64 years, 65–74 years, and ≥ 75 years), highest educational qualification (none, school, high school, trade, certificate or diploma, university), annual household income (0–$19.9 K, $20 K–$29.9 K, $30 K–$39.9 K, $40 K–$49.9 K, $50 K–$69.9 K, ≥ $70 K), region of birth (East Asia, Europe, India, Lebanon, Other), work status (employed, retired, unemployed, unpaid work, disabled, homemaker, other, e.g., study) and relationship status (in a couple, living alone).

## Results

6724 (8.4%) from 79,649 participants had psychological distress. Unadjusted prevalence was higher among people in apartments at 11.3% (1486/13,196) and lower among those in houses at 7.9% (5238/66,453). Prevalence was lower with more green space (Table 1). For example, comparing people with > 60% green space with those ≤ 20%, prevalence was 6.7% lower in houses and 3.2% lower in apartments. Bigger differences were observed for tree canopy. For example, the difference in prevalence between > 30% and < 10% tree canopy was 7.6% lower and 11.4% lower for people in houses and apartments, respectively. In contrast, prevalence was 3.2% and 8.8% higher among people with > 30% compared with < 10% open grass nearby houses and apartments, respectively. Further summaries of the study sample are provided in Appendixes 2 and 3.

**Table 1:** Patterning of high risk of psychological distress by green space variables and housing type

	Houses			Apartments		
	*N*	*n* high risk	% high risk	*N*	*n* high risk	% high risk
Total green space						
0–20%	877	110	12.5	798	110	13.8
21–40%	32,381	2871	8.9	9763	1083	11.1
41–60%	29,466	2041	6.9	2493	278	11.2
> 60%	3729	216	5.8	142	15	10.6
Tree canopy						
< 10%	8317	1043	12.5	1269	234	18.4
11–20%	25,815	2418	9.4	6295	820	13.0
21–30%	15,368	942	6.1	4169	329	7.9
> 30%	16,953	835	4.9	1463	103	7.0
Open grass						
< 10%	32,661	2051	6.3	9964	954	9.6
11–20%	18,702	1663	8.9	2118	331	15.6
21–30%	11,941	1224	10.3	973	175	18.0
> 30%	3149	300	9.5	141	26	18.4

High Risk Kessler 10 psychological distress scale scores >  = 22 / 50

**Fig. 1:**
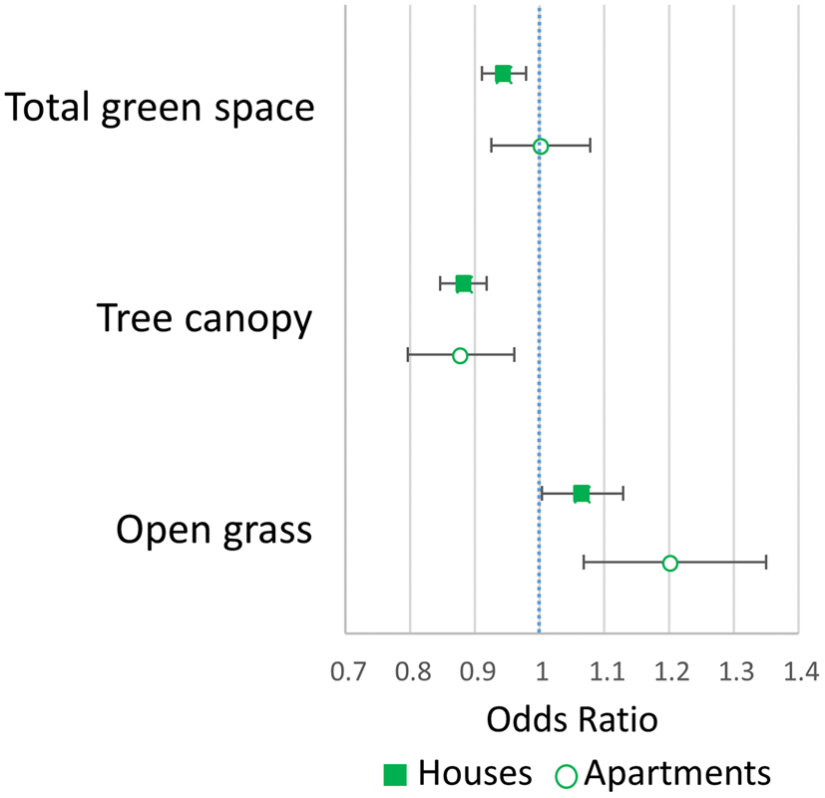
Associations between a 10% increase in total green space, tree canopy, and open grass, with high risk of psychological distress, stratified by housing type and adjusted for age, sex, income, education, work status, relationship status, and region of birth

Adjustment for confounding explained association between green space and psychological distress (Fig. 1). A 10% increase in green space was associated with 0.94 (95% CI = 0.91–0.98) lower odds of psychological distress among people in houses. 10% increases in tree canopy were associated with 0.88 (95% CI = 0.85–0.92) and 0.87 (95% CI = 0.79–0.96) lower odds of psychological distress among people in houses and apartments, respectively. A 10% increase in open grass was associated with 1.06 (95% CI = 1.00–1.13) and 1.20 (95% CI = 1.07–1.35) higher odds of psychological distress among people in houses and apartments, respectively.

## Discussion

This study indicates urban greening strategies focussed on increasing tree canopy cover in cities may support better mental health among people living in houses or apartments. These results align with work that reported benefits contingent on green space type [16–18]. They highlight the need to ensure the preservation and restoration of tree canopy cover, especially in high density communities, where apartments are common.

These results also clearly demonstrate that studies which focus on overall green space available could erroneously conclude that there is no mental health benefit for apartment-dwellers with more green space nearby overall. This finding was due to the increased levels of psychological distress coinciding with more open grass, with apartment-dwellers seemingly most affected.

Although people living in apartments in our study tended to have poorer mental health compared to their peers living in houses (a finding in accordance with other studies [15]), we found no evidence they had disproportionate benefits from green space per se, or by green space type. This does not support the ‘equigenesis’ hypothesis, [13] wherein health inequities might be potentially narrowed as a result of people in more disadvantaged circumstances benefiting more from green space than well-resourced and socially advantaged counterparts. If anything, the stronger positive (i.e., not healthy) association between open grass and psychological distress among people in apartments may indicate the opposite. These findings indicate that there may be factors that constrain or outweigh the accrual of psychological benefits from open grass, resulting in a net-negative for mental health. This may be due to mismatch between preferences and on-the-ground realities, lower levels of biodiversity, etc. It may also be related to accessibility, as areas with more open grass may also be more sprawling with private gardens and less connected or walkable, meaning people spend more time in cars for errands over short distances, rather than enjoying light exercise and serendipitous meetings with neighbours in nearby green spaces while en-route [9]. In contrast, areas with greater tree canopy cover offer enhanced opportunities for being outdoors, walking and more vigorous physical activity in these cities due to provision of shade, since the urban heat island effect is common, as well as proximity to resident wildlife and aesthetic pleasure. [35].

Our findings cannot be interpreted in causal terms due to cross-sectional design. Selection into houses over apartments and vice versa is not random and for reasons correlated with green space availability and factors that influence mental health. Green land-use may also have decreased over time in some areas relative to when the survey was conducted, making our estimate of green space availability conservative. The 45 and Up Study had a low response rate (18%), though a demographic profile that was generally representative of the Australian population aged > 45y [36].

There may be multiple mediating processes operating in serial or parallel that contribute to our findings, including physical activity, sleep, social connection and restoration. Assessment of mediation was beyond the scope of this article but warrants dedicated attention in future work, especially since the mediators that link green space with mental health among occupants of houses may be different to those living in apartments.

Overall, investments in tree canopy may benefit the mental health of house and apartment residents relatively equally. Further work is needed to understand factors constraining the prevention potential of open grass, to unlock and maximise its benefits for mental health.

## Data Availability

The 45 and Up Study is available to researchers via application to the Sax Institute for a data user licence.

## Appendix 1

### Kessler 10 psychological distress items in the 45 and up study

During the past 4 weeks, about how often did you feel:

- Tired out for no good reason?
- Nervous?
- So nervous that nothing could calm you down?
- Hopeless?
- Restless or fidgety?
- So restless that you could not sit still?
- Depressed?
- That everything was an effort?
- So sad that nothing could cheer you up?
- Worthless?
- None of the time
- A little of the time
- Some of the time
- Most of the time
- All of the time

## Appendix 2

See Table 2.

**Table 2:** Summary of overall sample

	*N*	%
Total	82,937	8.6
Current housing		
House	66,453	7.88
Apartment	13,196	11.26
Retirement village	1581	7.34
Hostel	286	17.13
Mobile home	201	11.44
Other	644	22.97
Not stated	576	12.85
Total green space		
0–20%	1741	13.61
21–40%	43,904	9.57
41–60%	33,207	7.38
≥ 60%	4085	6.02
Tree canopy		
< 10%	9992	13.68
11–20%	33,452	10.22
21–30%	20,355	6.67
≥ 30%	19,138	5.18
Open grass		
< 10%	44,297	7.22
11–20%	21,800	9.75
21–30%	13,389	10.94
≥ 30%	3451	10.00

## Appendix 3

See Table 3.

**Table 3:** Summary of overall sample, by psychological distress status

	K10 < 22		K10 ≥ 22	
	*N*	%	*N*	%
Overall	75,803	91.4	7134	8.6
	Mean	SD	Mean	SD
Total green space	39.15	12.1	36.83	11.73
Tree canopy	22.16	11.24	18.44	10.1
Open grass	12.39	8.25	13.81	8.4
Age	62.07	11.18	60	11.46

	*N*	%	*N*	%
Female	38,975	51.4	3453	48.4
Income < $70 K	34,251	45.18	3434	48.14
Education < university	50,564	66.7	4472	62.69
Living alone	17,910	23.63	2171	30.43

*K10* Kessler 10 psychological distress scale, *SD* standard deviation
